# ComOn-Coaching: The effect of a varied number of coaching sessions on transfer into clinical practice following communication skills training in oncology: Results of a randomized controlled trial

**DOI:** 10.1371/journal.pone.0205315

**Published:** 2018-10-05

**Authors:** Marcelo Niglio de Figueiredo, Lorena Krippeit, Gabriele Ihorst, Heribert Sattel, Carma L. Bylund, Andreas Joos, Jürgen Bengel, Claas Lahmann, Kurt Fritzsche, Alexander Wuensch

**Affiliations:** 1 Department of Psychosomatic Medicine and Psychotherapy, Medical Center – University of Freiburg, Faculty of Medicine, University of Freiburg, Freiburg, Germany; 2 Clinic of Dermatology and Venereology, Medical Center – University of Freiburg, Faculty of Medicine, University of Freiburg, Freiburg, Germany; 3 Clinical Trials Unit, Medical Center – University of Freiburg, Faculty of Medicine, University of Freiburg, Freiburg, Germany; 4 Psychosomatic Medicine and Psychotherapy, Klinikum rechts der Isar, Technical University of Munich, Munich, Germany; 5 College of Journalism and Communications, College of Medicine, University of Florida, Gainesville, Florida, United States of America; 6 Institute of Psychology, Rehabilitation Psychology and Psychotherapy, University of Freiburg, Freiburg, Germany; University of Newcastle, AUSTRALIA

## Abstract

**Objective:**

To investigate the effect of the number of coaching sessions after communication skills training on the medical communicative performance of oncologists in clinical practice.

**Methods/Design:**

The training, consisting of a workshop and one (control group) vs. four (intervention group) sessions of individual coaching, was evaluated in a randomized controlled trial. Eligible participants included physicians working in any setting where patients with oncological diseases were treated. Real medical consultations were video recorded at three time points: before the workshop (t0), after the workshop (t1) and after completion of coaching (t2). The 1.5-day workshop was based on role-playing in small groups; in the coaching sessions, the videos recorded at t1 were analyzed in detail by both the trainer and the physician. The coaching sessions were manualized and based on the physician’s learning goals. The primary hypothesis was that the intervention group would improve to a higher extent than the control group, as assessed by external raters using rating scales specially developed for this project. Physicians were stratified for sex and setting and randomized by an independent statistician. The group assignment was revealed for physicians and trainers at the end of the workshop, while the raters were blinded to group assignments and assessment points.

**Results:**

A total of 72 physicians participated in one of 8 workshops and could be allocated to either the control or intervention group. The intervention group showed a statistically significant improvement (ES d = 0.41, p<.01) in the *All items* domain of the rating scales between t1 and t2 and showed a significant advantage compared with the CG (ES = .41, p = .04). The impact on diverse specified skills was heterogeneous; a larger sample is necessary for more detailed analysis.

**Conclusions:**

The training achieved some observable and significant changes in the communicative behavior of oncologists in clinical practice. The four coaching sessions showed some significant advantages compared to the single coaching session. Considerable effort is necessary to achieve sustained changes in communication in clinical every-day practice. Thus, our coaching concept is a promising method for this purpose.

## Background

Research on communication training for physicians and other health care professionals (HCP), particularly in oncology [1–3], is experiencing a transition. The research in the last fifty years has stressed the primordial role of communication in health care in general and particularly in the medical work [4,5], stressing the meaning of the physician-patient communication for the treatment outcome [6]. The implementation of various communication skills training programs [2,7–15], effective assessment instruments [7,14,16–18] and advanced training methods [19–21] that showed demonstrable positive effects has led the debate on communication in medicine to a higher level: concepts are being redefined [22–28], old-established models are being enhanced [29], new models are being developed [30,31], and a single workshop is being complemented or replaced by shorter interventions (booster sessions, supervision) spread over a longer training period [30,32–38]. A crucial question remains how to transfer skills into clinical practice [39]. Studies have shown that the effects of the training are minor when assessed with real patients and do not last long, the effect disappearing after no later than one year [2,7,34,40]. This problem of low transfer despite intensive work is known from Organization Psychology [41] and has triggered significant research, the aim of which is to better understand how transfer works and identify which factors support or disturb this process [25,42–44]. Several problems have been identified that hamper the medical transfer in the clinical practice [39,45]. Physicians often lack formal CST during clerkships and the clinician teachers lack skills for teaching communication. Moreover, the training environment is characterized by a learning culture where communication skills are seldom addressed and the content of the consultations are normally considered more important than the interaction between clinician and patient. Furthermore, strongly hierarchical work cultures, such as the medical one in many places, reinforce the self-consciousness of the trainees and disturb the learning process. Finally, practitioners have often difficulty in understanding the relationship between theoretical communication models and the realities of clinical practice. A number of methods have been suggested to meet these challenges such as the work with self-defined goals [46], structured feedback [47], the use of video based feedback [34] and the use of role playing with actor-patients in small groups [47].

Therefore, the goal of the present study was to facilitate the transfer of learned communication skills in oncology into clinical practice. For this purpose, we built upon a previous work in which we developed a communication skills training (CST) consisting of a workshop and one subsequent individual coaching session (ComOn CST). This previous training was evaluated with standardized patients for specific situations, such as the transition from curative to palliative [48,49] and the discussion of information about randomized controlled trials [50,51]. The content of the new training was expanded so that consultations including various types of physician-patient-conversations in oncology would be considered. To foster transfer, the coaching concept was thoroughly elaborated and manualized. Therefore, the aim of the present study was to determine the effect of enhanced coaching in an evaluation study using actual consultations.

## Objectives

The main purpose of the present study is to determine the influence of the number of coaching sessions (one vs. four) on the efficacy of the ComOn CST. Efficacy was evaluated in real consultations, using the ComOn Rating Scale (ComOn RS).

**Primary hypothesis**: After a CST workshop, participants in the enhanced four session coaching condition (intervention group) will have significantly better communication skills performance than the participants in the one session coaching (control group, as previously evaluated).

**Intermediate hypothesis**
1. The training (workshop plus coaching) will show significant changes for both IG and CG, but at least for the IG. This should confirm that the original training is effective also with real patients.
2. As the workshop was the same for both IG and CG, it will be checked if both groups differ one from another after the workshop (baseline for the calculation of the effect of the coaching). No significant changes are expected.
3. Significant changes between before and after the coaching are expected at least for the IG.

As the intermediate research questions are prerequisites for the main one, the former will be presented first in the results.

## Methods/Design

### Trial design

**Fig 1:**
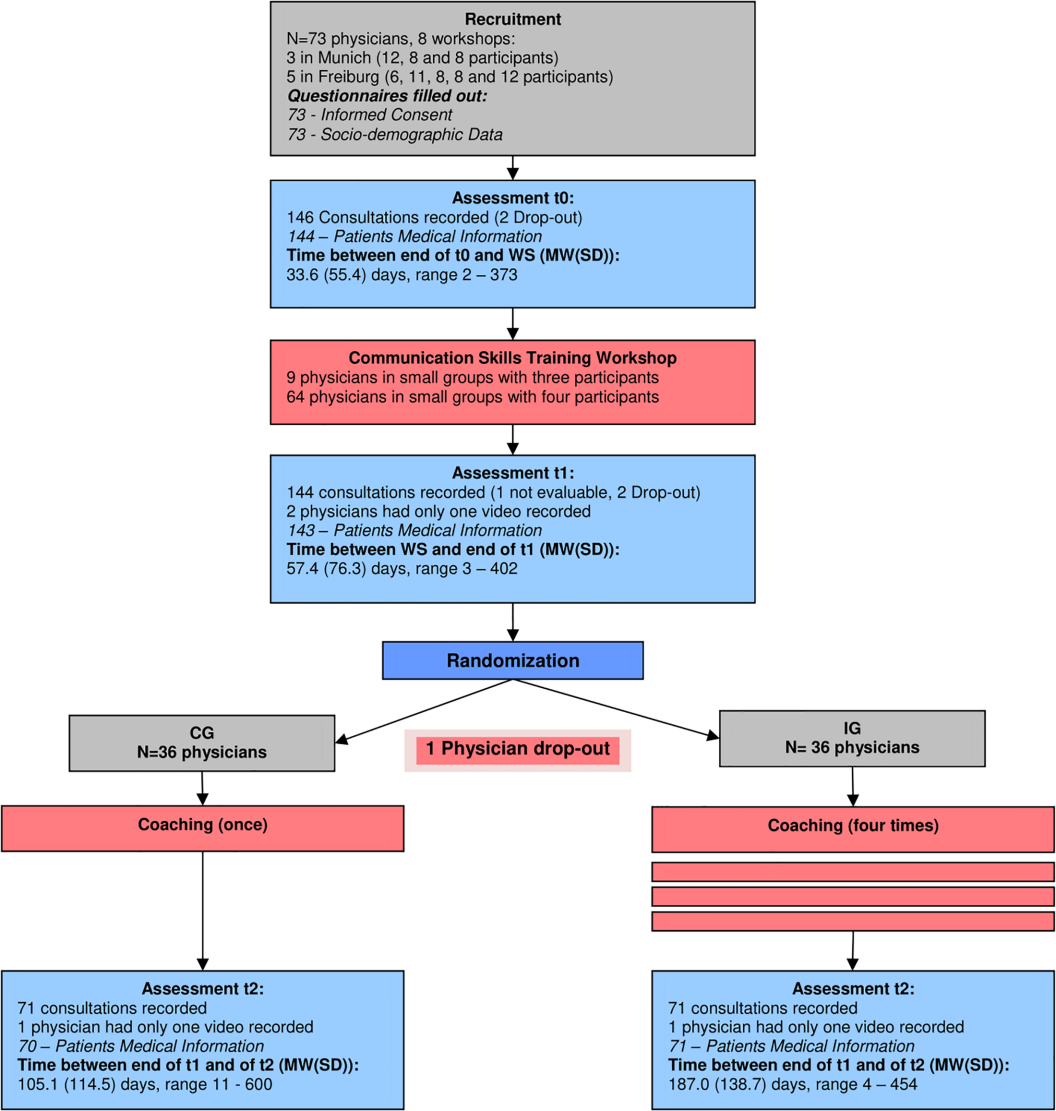
Study design.

The randomized controlled trial was conducted in Freiburg and Munich, Germany. Fig 1 shows the study design and the actual data collected. Before the workshop, each physician obtained video-recordings of two real consultations with patients during their daily practice (assessment point t0). The physicians selected and invited patients among those who they were currently treating. We only recorded consultations with cancer patients who, after being informed about the study, gave their written consent. The consultations were open to all themes linked to cancer treatment and included breaking bad news, treatment discussion, delivery of medical information, regular control consultations etc. After the workshop, two parallel and even-numbered groups of participating physicians were established, receiving a different number of coaching sessions (Intervention Group (IG): four sessions; Control Group (CG): one session). Each physician recorded two additional consultations before the first coaching session (t1). After the completion of coaching (the number of sessions depending on the group assignment), two more consultations were video-recorded by each physician (t2). The first video recording was made in July 2013 and the last recording was made in January 2016.

### Participants

Physicians working in the field of oncology were eligible to participate in the present study. Participation was voluntary though attractive due to the ability to earn continuing education credits. Physicians were asked by AW and MNF to participate and to recommend their colleagues for recruitment using a snowball recruitment method. The recruitment was closed when 72 physicians registered for the training. One physician in the first workshop did not continue after assessment t1 and was substituted by another physician in a subsequent workshop. Thus, a total of 73 physicians participated in eight workshops between July 2013 and June 2015, and 72 complete data sets were assessed and analyzed. Three participants did not come from the study centers or affiliated clinical institutions. These individuals participated in the workshop and were coached via Skype by AW.

### Intervention

The 1.5-day workshop was manualized and based on the workshop developed and evaluated in previous studies [48,51]. The workshop consisted of a short theoretical introduction followed by work in small groups of four physicians. In the small groups each physician could practice on challenging situations of own practice in role plays with actor patients. The coaching concept was manualized based on the Miller Pyramid [52] and the self-regulation model for behavioral changes [53]. The coaching consisted of four sessions: in the first two sessions, the videos recorded at t1 were analyzed by the trainer and the physician together; in the third session, one critical passage of the videos was discussed in detail; and the fourth session was conceptualized as an open supervision. The control group had only one session, where one of the videos was analyzed. Each small group had the same trainer throughout the workshop and in the coaching sessions. The theoretical background of the training was based on the SPIKES-Modell [54]. The ComOn RS, the instrument used for the evaluation of the training, was developed to match the taught skills [55], so that its items (see *Outcomes* below) describe also taught content. Table 1 shows the main characteristics of the study. Additional details are provided in the study protocol [56].

**Table 1:** Study characteristics.

**Participants**	
Physicians	Inclusion: work with cancer patients
	written consent for the study
Patients	Inclusion: consultation about cancer (diagnosis, treatment, control, etc.)
	written consent for the study
**Training**	
Workshop	1.5-days (12.75 h), based on Goelz et al. (2010) and Wuensch et al. (2010)
Coaching	IG: four manualized sessions
	CG: one manualized session (analogous to Goelz et al. 2010 and Wuensch et al. 2010)
**Instruments**	
External Raters	ComOn-Coaching Rating Scales (Niglio de Figueiredo et al. 2017)
Physicians	Sociodemographic data
	Communicative competence self-evaluation (Items corresponding to Rating scales)
	Expectation for the consultation
	Self-evaluation of the consultation (Items corresponding to Rating scales)
Patients	Sociodemographic data
	Medical information (filled out by physician)
	Expectation for the consultation
	Evaluation of the consultation (Items corresponding to Rating scales)

### Randomization

When participants were registered for the workshop, small groups of four physicians were established, two of which were randomly allocated into one of the treatment groups. This strategy enabled us to ensure that all trainers had trained both IG and CG. The allocation was based on a computer-generated list of random numbers and executed by an independent statistician (HS). In addition, the participants were stratified for sex and occupational setting (inpatient or outpatient). As blinding was not possible for the entire process, the group assignment was concealed to trainers and participants using opaque and sealed envelopes until the end of the workshop.

### Trainers

A total of 12 trainers were involved in the project. All trainers were psychologists/physicians with proficiency in communication training. Six of the trainers had training experience and were involved in the conceptual development of the training. The other trainers were trained by the senior author (AW) and assisted in the training prior to working as independent trainers.

### Outcomes

Each consultation was evaluated by one of two raters who were blinded to group assignments and assessment points. The raters, two psychologists with experience in consultations with cancer patients, were trained in using the ComOn-Coaching Rating Scales [57] for both the validation study of the instrument and the present evaluation study. The training consisted of eight sessions of 3-4h each, where recorded consultations were rated and discussed till acceptable agreement levels were achieved. Table 2 shows the items of the ComOn-Coaching Rating Scales and the interrater-reliability (given as interclass correlation, ICC) achieved.

**Table 2:** Checklist ComOn-Coaching. (Assessment in a 5-point scale, 0–4).

Subjective global evaluation		ICC
How do you assess the communicative competence of the physician in this conversation?		
**A1**	**Start of the conversation**	**.44**
A1	Does the physician initiate the conversation appropriately?	
**A2**	**Patient’s Perspective**	**.49**
A2	Does the physician manage to get an idea of the patient’s perspective at the beginning of, or during the consultation?	
**B**	**Structure of conversation**	**.64**
B1	Does the physician actively give structure to the conversation (set an agenda of central topics)?	.67
B2	Does the physician set sub-sections in the course of conversation (in detail)?	.46
**C**	**Emotional issues**	**.54**
C1	Does the physician recognize the patient´s emotions and does he do they name them; evaluation based on NURSE by Back (2008)	.59
C2	Does the physician offer emotional support?	.43
**D**	**End of conversation**	**.42**
D1	Does the physician summarize the content of the consultation and do they close the conversation appropriately?	
**E**	**General communication skills**	**.70**
E1	Does the physician use clear and appropriate words during the conversation?	.41
E2	Does the physician use appropriate non-verbal communication during the consultation?	.61
E3	Does the physician adjust his pace during the consultation and does he make appropriate pauses?	.38
E4	Does the physician offer the patient the chance to ask questions during the consultation?	.88
E5	Does the physician check whether the patient has understood the consultation?	.70
**F**	**Overall Evaluation**	**.35**
F1	How do you assess the communication skills of the physician in this conversation?	
	**All Items**	**.66**
**Suggested interpretation of the ICC: <0.2 = poor, 0.21–0.40 = fair, 0.41–0.60 = moderate, 0.61–0.80 = good, 0.81–1.00 = very good.**		

As the interrater-reliability was only moderate for some items, the rater effect was considered as a covariate for the calculation, and the item with the lowest reliability *Overall evaluation* (Item F) was substituted by the average of all items (*All items*), which showed good reliability. The items are summarized as areas/domains, which are indicated in bold. In this article, all results are shown as summarized domains. The results for the single items are provided in the supporting information.

### Sample size

The sample size calculation was performed based on effects sizes ranging between 0.61 and 0.78 observed in the previous project [49]. Therefore, we aimed at demonstrating a significant difference between IG and CG with a power of 80% when an effect size of ES = 0.7 is assumed. The sample size calculation was based on the two-sample t-test at a two-sided level (α = 5%) and resulted in 34 physicians per group. We increased the size of each group by 5% to account for potential dropouts, for whom no data enabling an intention-to-treat analysis can be obtained. Therefore, we planned to randomize 72 physicians.

### Statistical methods

Statistical analysis was conducted with SAS 9.2 (SAS Institute Inc., Cary, NC, USA) by an independent statistician (GI). As the only physician with incomplete data was substituted by another physician, we did not perform any imputation strategies to account for this dropout.

We used descriptive methods to analyze baseline characteristics and changes in evaluation compared to baseline. Paired t-tests were applied to assess changes from baseline, as visual inspection of the data justified the assumption of normally distributed data.

The hypotheses regarding the additional benefit of intensive coaching were investigated with linear mixed regression models. Mixed regression models may incorporate fixed effects (e.g. treatment group) as well as random effects (e.g. rater) and repeated measurements [58]. Group comparisons (IG vs. CG) at t2 of the evaluation by the external raters were analyzed with these models. The models account for repeated measurements due to two consultations for each physician at each time point, control for baseline evaluation (averaged over two consultations), patient distress, and a rater random effect. Effect sizes were derived using the estimated treatment effect and the estimated standard deviation of the observations obtained from the mixed models.

### Ethical issues

This study was approved by the ethics committees of the University Medical Center Freiburg, Freiburg, Germany, and the University Hospital *Klinikum rechts der Isar*, Munich, Germany, and is registered under DRKS00004385 in the DRKS (German Clinical Trials Register). Physicians and patients were informed verbally and in writing and gave their written informed consent.

## Results

### Samples

Table 3 describes the physician sample. The physicians were 34 years old on average (SD = 8.1) and had a mean of 6 years work and 3 years oncological experience. Five physicians were in training for medical specialization, and 17 physicians were already specialists (i.e., licensed to work as senior physicians or in private practice). Twenty-two physicians worked in Hematological Oncology, 12 physicians worked in other internal medicine specialties, and 15 physicians worked in Oncological Gynecology. The IG and CG did not differ in any of the assessed variables to a relevant extent.

**Table 3:** Physician sample.

		**All (%)**		**Intervention Group**		**Control Group**	
**N**		72 (100%)		36 (100%)		36 (100%)	
**Sex**	Male	25 (34,7%)		12 (33.3%)		14 (38.9%)	
	Female	47 (65,3%)		24 (66.7%)		22 (61.1%)	
**Medical Specialist**	Yes	17 (23.6%)		7 (19.4%)		10 (27.8%)	
	No	55 (76.4%)		29 (80.5%)		26 (72.2%)	
**Area of Specialization**	Hemato-Oncology	22 (30.6%)		13 (36.1%)		9 (25%)	
	Gynecology	15 (20.8%)		9 (25.0%)		6 (16.7%)	
	Internal Medicine (other)	12 (16.7%)		5 (13.9%)		7 (19.4%)	
	Radiology	9 (12.5%)		2 (5.6%)		7 (19.4%)	
	Neurosurgery	3 (4.2%)		1 (2.8%)		2 (5.6%)	
	Anesthesia/Pall. med.	3 (4.2%)		1 (2.8%)		2 (5.6%)	
	Pediatric	2 (2.8%)		1 (2.8%)		1 (2.8%)	
	Ear, Nose and Throat	2 (2.8%)		1 (2.8%)		1 (2.8%)	
	Other	4 (5.6%)		3 (8.3%)		1 (2,8%)	
		**All**		**IG**		**CG**	
		**N**	**Mean (SD)**	**N**	**Mean (SD)**	**N**	**Mean (SD)**
**Age**		72	33,8 (8,1)	36	33,8 (8,0)	36	34,0 (8,0)
**Professional experience (years)**		72	6,0 (7,2)	36	6,0 (7,5)	36	6,0 (6,9)
**Experience in oncology (years)**		70	2,8 (4,3)	36	3,3 (5,2)	34	2,3 (3,0)
**Percent oncological patients**		72	73,7 (33,0)	37	69,8 (36,4)	35	78 (29,0)
**Comm. courses (hours)**		68	6,3 (18,1)	35	7,7 (19,6)	33	5,0 (17,0)
**Comm. Workshops (hours)**		69	4,3 (11,7)	33	2,4 (5,8)	36	6 (15,0)

Tables 4 and 5 describe the patient sample. A total of 428 consultations with different patients were recorded.

**Table 4:** Patient sample I.

Physician’s Group		All	Intervention Group				Control Group			
Assessment Point		All	All	t0	t1	t2	All	t0	t1	t2
Treatment Status (physician’s answer)	Palliative	140	66	19	21	26	74	24	31	19
	Curative	208	102	37	34	31	106	40	28	38
	Unclear	53	34	11	13	10	19	6	7	6
	n/r	24	13	5	4	4	11	1	4	6
Gender	Male	198	103	37	39	27	95	39	25	31
	Female	226	115	34	33	44	115	32	45	38
	n/r	1	1	1	0	0	0	0	0	0
Nationality	German	390	197	63	65	69	193	63	65	65
	Other	32	15	6	7	2	17	8	5	4
	n/r	3	3	3	0	0	0	0	0	0
Permanent relationship?	Yes	293	156	51	55	50	137	53	39	45
	No	124	54	17	17	20	70	17	31	22
	n/d	8	5	5	0	0	3	1	0	2
Children?	Yes	318	158	52	54	52	160	55	52	53
	No	104	55	19	18	18	49	16	17	16
	n/d	3	2	1	0	1	1	0	1	0
Living conditions	Alone	104	47	16	16	15	57	16	26	15
	With partner	195	100	31	34	35	95	32	30	33
	With partner and children	83	49	18	18	13	34	17	7	10
	Alone with children	19	6	2	1	3	13	2	4	7
	With the parents	13	6	1	2	3	7	3	1	3
	Other	9	5	3	0	2	4	1	2	1
	n/r	2	2	1	1	0	0	0	0	0

**Table 5:** Patient sample II.

Physician’s Group		All	Intervention Group				Control Group			
Assessment Point		All	All	t0	t1	t2	All	t0	t1	t2
Occupational Status	working	58	35	12	8	15	23	9	6	8
	in illness license	124	66	22	27	17	58	25	14	19
	not working/ in pension	239	110	36	37	37	129	37	50	42
	n/r	4	4	2	0	2	0	0	0	0
Highest educational achievement	Secondary school (9th grade)	136	69	29	20	20	67	22	30	15
	Middle school (10th grade)	137	66	16	27	23	71	19	18	34
	Baccalaureate (12th grade)	47	27	12	8	7	20	8	7	5
	University	92	46	10	17	19	46	19	14	13
	n/r	13	7	5	0	2	6	3	1	2
Disease status (physician’s answer)	First tumor	270	135	47	48	40	135	45	44	46
	Second tumor	16	9	3	5	1	7	3	2	2
	Relapse	77	33	11	11	11	44	16	16	12
	Remission	33	24	7	5	12	9	5	1	3
	Unclear	8	2	1	0	1	6	2	2	2
	Unknown	5	2	0	1	1	3	0	3	0
	n/r	16	10	3	2	5	6	0	2	4
Metastasis? (physician’s answer)	Yes	123	55	19	18	18	68	20	24	24
	No	193	99	40	27	32	94	40	28	26
	Unknown	37	21	2	10	9	16	4	6	6
	n/r	72	40	11	17	12	32	7	12	13
Age	N	421	211	68	72	71	210	71	70	69
	Mean (SD)	58,88 (15,49)	57,59 (16,26)	58,40 (15,35)	57,29 (16,81)	57,13 (16,75)	60,18 (16,61)	57,62 (14,91)	62,43 (14,31)	60,52 (14,42)
Distress	N	418	209	70	70	69	209	70	70	69
	Mean (SD)	48,27 (29,64)	47,73 (29,23)	41,83 (29,02)	49,59 (28,71)	51,84 (29,42)	48,81 (30,12)	52,14 (29,95)	55,31 (28,95)	38,84 (29,29)

The average age of the patients was 59 years (SD = 15.51); two conversations were conducted with the patient’s parents (1 and 10 y. o.); 49% of the patients received curative treatment, and 33% of the patients received palliative treatment according to the treating physician’s declaration of. Among all the assessed variables, patients of the IG and the CG noticeably differed only in distress over the three assessment points, with the physicians of the IG having significantly more distressed patients at t2. Therefore, this variable was used as a covariate for the mixed models calculation.

### Intermediate hypothesis I: Effect of the training

As stated above, the first hypothesis tested was whether the present training concept (workshop plus coaching) was effective with real patients. Table 6 shows the assessment of the consultations by the external raters at t0 (before workshop) and t2 (after coaching), and the changes between t0 and t2 for both groups. Significant effects (marked **bold** in the table) were achieved by both the IG (*Start of conversation* (p = 0.0004), *General communication skills* (p = 0.0025) and *All items* (p = 0.0064)) and the CG (*Start of conversation* (p = 0.0126) and *Structure of Consultation* (p = 0.0153)). These results suggest that the training concept was able to produce changes on the behavior of the physicians as expected.

**Table 6:** Effect of the training.

Variable	Group	Mean t0 (SD)	Mean t2 (SD)	Diff (SD)	Effect Size	P
**A1 Start of Consultation**	**IG**	**1.79 (0.70)**	**2.27 (0.75)**	**0.48 (0.72)**	**0,67**	**0.0004**
	**CG**	**1.68 (0.66)**	**2.07 (0.70)**	**0.39 (0.89)**	**0,44**	**0.0126**
	***All***	***1*.*74 (0*.*68)***	***2*.*17 (0*.*73)***	***0*.*43 (0*.*80)***	***0*,*54***	***<0*.*0001***
**A2 Assessing Patient’s Perspective**	IG	2.33 (0.90)	2.64 (0.75)	0.31 (1.02)	0,30	0.0750
	CG	2.23 (0.86)	2.14 (0.93)	-0.09 (1.19)	-0,08	0.6521
	*All*	*2*.*28 (0*.*88)*	*2*.*39 (0*.*88)*	*0*.*11 (1*.*12)*	*0*,*10*	*0*.*4029*
**B Structure of Consultation**	IG	2.39 (0.72)	2.47 (0.74)	0.08 (1.06)	0,08	0.6391
	**CG**	**2.07 (0.53)**	**2.42 (0.68)**	**0.35 (0.82)**	**0,43**	**0.0153**
	*All*	*2*.*23 (0*.*65)*	*2*.*45 (0*.*71)*	*0*.*22 (0*.*95)*	*0*,*23*	*0*.*0578*
**C Emotional Issues**	IG	2.36 (0.83)	2.64 (0.74)	0.28 (1.09)	0,26	0.1307
	CG	2.29 (0.73)	2.34 (0.79)	0.06 (0.82)	0,00	0.6859
	*All*	*2*.*32 (0*.*77)*	*2*.*49 (0*.*78)*	*0*.*17 (0*.*96)*	*0*,*18*	*0*.*1425*
**D End of Consultation**	IG	1.75 (0.79)	2.02 (0.87)	0.27 (1.04)	0,26	0.1372
	CG	1.69 (0.65)	1.82 (0.81)	0.13 (1.06)	0,12	0.4777
	*All*	*1*.*72 (0*.*72)*	*1*.*92 (0*.*84)*	*0*.*20 (1*.*05)*	*0*,*19*	*0*.*1180*
**E General communication skills**	**IG**	**2.59 (0.47)**	**2.84 (0.38)**	**0.26 (0.48)**	**0,54**	**0.0025**
	CG	2.47 (0.33)	2.61 (0.47)	0.14 (0.55)	0,25	0.1488
	***All***	***2*.*53 (0*.*41)***	***2*.*73 (0*.*44)***	***0*.*20 (0*.*52)***	***0*,*38***	***0*.*0018***
**F Overall Evaluation**	IG	2.53 (0.86)	2.68 (0.67)	0.15 (0.96)	0,16	0.3482
	CG	2.40 (0.60)	2.49 (0.79)	0.09 (0.87)	0,10	0.5387
	*All*	*2*.*47 (0*.*74)*	*2*.*59 (0*.*74)*	*0*.*12 (0*.*91)*	*0*,*13*	*0*.*2628*
**All items**	**IG**	**2.40 (0.44)**	**2.62 (0.40)**	**0.23 (0.47)**	**0,49**	**0.0064**
	CG	2.24 (0.34)	2.40 (0.49)	0.16 (0.58)	0,28	0.1050
	***All***	***2*.*32 (0*.*40)***	***2*.*51 (0*.*46)***	***0*.*19 (0*.*53)***	***0*,*36***	**0.0026**

Evaluation of the consultations of all physicians by external raters at t0 and t2 (scale range: 0–4); p-value from paired t-test to assess differences between t0 and t2.

### Intermediate hypothesis II: Effect of the workshop

Before we discuss the effect of coaching on the groups, the effect of the workshop alone should be considered (S1 Table): no significant effect was achieved for either of the groups alone (p-range 0.12–0.91) or both groups together (p-range 0.06–0.92), so that both groups does not differ significantly one from another after the workshop, and neither differs significantly from before the workshop. Some non-significant changes seem to be important to understand the effect of the coaching, as will be discussed below.

### Intermediate hypothesis III: Effect of the coaching on the groups

The second step is to test the hypothesis that the coaching would have a significant effect on the communicative behavior of the physicians. Table 7 shows the assessment of the consultations by the external raters at t1 (before coaching) and t2 (after coaching) and the changes between t1 and t2 for both groups.

**Table 7:** Effect of the coaching on the groups.

Variable (Domain)	Group	Mean t1 (SD)	Mean t2 (SD)	Diff (SD)	Effect Size	P-value
**A1 Start of Conversation**	**IG**	**1.98 (0.72)**	**2.27 (0.75)**	**0.29 (0.85)**	**0,36**	**0.0482**
	**CG**	**1.79 (0.60)**	**2.07 (0.70)**	**0.28 (0.79)**	**0,34**	**0.0416**
**A2 Assessing Patient’s Perspective**	**IG**	**2.22 (0.86)**	**2.64 (0.75)**	**0.42 (0.82)**	**0,35**	**0.0044**
	CG	2.19 (0.92)	2.14 (0.93)	-.06 (1.06)	0,51	0.7545
**B Structure of Conversation**	IG	2.34 (0.60)	2.47 (0.84)	0.14 (0.74)	-0,06	0.2782
	**CG**	**2.15 (0.56)**	**2.42 (0.68)**	**0.27 (0.76)**	**0,19**	**0.0368**
**C Emotional Issues**	IG	0.50 (0.67)	2.64 (0.74)	0.14 (0.78)	0,36	0.2938
	CG	2.43 (0.83)	2.34 (0.79)	-.08 (0.83)	0,18	0.5496
**D End of Conversation**	IG	2.02 (0.83)	2.02 (0.87)	0.00 (1.05)	-0,10	1.0000
	CG	1.84 (0.66)	1.87 (0.85)	0.03 (1.07)	0,00	0.8768
**E General Communication skills**	**IG**	**2.67 (0.49)**	**2.84 (0.38)**	**0.18 (0.45)**	**0,03**	**0.0266**
	CG	2.61 (0.41)	2.61 (0.47)	0.00 (0.43)	0,40	0.9691
**F Overall Evaluation**	IG	2.60 (0.59)	2.69 (0.67)	0.09 (0.72)	0,00	0.4569
	CG	2.44 (0.61)	2.49 (0.79)	0.05 (0.79)	0,13	0.7150
**All items**	**IG**	**2.44 (0.43)**	**2.62 (0.40)**	**0.18 (0.40)**	**0,06**	**0.0102**
	CG	2.35 (0.36)	2.40 (0.49)	0.05 (0.45)	0,45	0.5110

Evaluation of the consultations by external raters at t1 and t2 (scale range: 0–4) separately by treatment group; p-value from paired t-test to assess differences between t1 and t2

The IG achieved a significant change in the areas *Start of conversation* (p = 0.0482), *Assessing patient’s perception* (p = 0.0044), *General communication skills* (p = 0.0266) and *All items* (p = 0.0102). The CG achieved a significant development in the areas *Start of conversation* (p = 0.0416) and *Structure of conversation* (p = 0.0368). The two coaching models had thus some significant influence on some domains.

### Primary hypothesis: Effect of the coaching—Comparison between the groups

In a last step, we addressed our main hypothesis that more intensive coaching would show a significant advantage compared to less intensive coaching. Table 8 shows the results from the mixed regression models investigating the comparison of the IG to the CG at t2, adjusting for baseline t1, patient distress, and rater. The IG shows a significant greater effect than the CG in the domains *Assessing patient’s perspective* (p = 0.0084), *General communication skills* (p = 0.0063) and *All items* (p = 0.0446). These differences show a medium effect size (0.41–0.51).

**Table 8:** Effect of the coaching—Comparison between the groups.

	Group difference IG minus CG				
Variable	Parameter Estimate	95% CI	Effect Size	Stand. Error	P
**A1 Start of Conversation**	0.16	-.15 to 0.47	0.19	0.16	0.3029
**A2 Assessing Patient’s Perspective**	**0.50**	**0.13 to 0.86**	**0.51**	**0.19**	**0.0084**
**B Structure of Conversation**	-.04	-.36 to 0.29	-.04	0.16	0.8223
**C Emotional Issues**	0.26	-.07 to 0.59	0.28	0.17	0.1273
**D End of Conversation**	0.10	-.31 to 0.51	0.10	0.21	0.6305
**E General Communication Skills**	**0.25**	**0.07 to 0.44**	**0.54**	**0.10**	**0.0063**
**F Overall Evaluation**	0.13	-.20 to 0.47	0.15	0.17	0.4247
**All items**	**0.19**	**0.00 to 0.38**	**0.41**	**0.10**	**0.0446**

Group Comparison of the difference IG minus CG, evaluation of the consultations by external raters at t2, adjusted in mixed regression models for baseline t1, rater, and patient distress. Parameter Estimates refer to the group difference IG minus CG at t2.

### Ancillary examination of process data

As both groups had a significant improvement in different domains through the workshop, a detailed examination of the changes through all three assessment points was undertaken in order to better understand these changes. For three items, the difference between the IG and CG values at t0 (before training) was larger than 0.2 points (pts), although not significant: B1 (Active structuring), B2 (Setting sub-sections), and E5 (Checking understanding). Among the B-items, there was practically no change in both groups between t0 and t1 (between workshop and coaching, see S1 Table):

B1: IG 2.8 pts (t0) and 2.77 pts (t1)
  CG 2.51 pts (t0) and 2.55 pts (t1);
B2: IG 1,92 pts (t0) and 1.88 pts (t1)
  CG 1.64 pts (t0) and 1.74 pts (t1).

Through coaching, the CG achieved a significant change and reached the same level as the IG (at t2 IG: 2.85 pts; CG 2.89 pts; see S3 Table). For the E5-item, both groups were markedly low at t0 (IG: 0.67 pts; CG: 0,42 pts) and achieved a positive change through the workshop (at t1 IG: 0.76 pts; CG: 0.61 pts) that became significant for both groups through coaching (at t2 IG: 1.02 pts; CG: 0.90 pts).

## Discussion

This randomized controlled trial (RCT) evaluated the effect of a different number of coaching sessions as part of an innovative training concept consisting of a workshop and coaching. After determining that the workshop alone did not produce changes by the physicians in real consultations, we were able to show that the whole training (workshop plus coaching) is able to produce such changes in both groups. In three out of eight domains the IG showed a significant greater effect.

While changes in the physicians’ behavior can be clearly achieved, these changes are very small and limited. The question goes thus beyond the simple question “is it worth adding three more coaching sessions?” It seems to imply that considerable changes can only be achieved by long and intensive work. The implication for the practice is that continuous interventions should be preferred to punctual ones. The implication for research is that further effort should be invested in evaluating CST and add-on interventions in real settings, where the effect sizes tend to be smaller.

Our training achieved significant effects in some domains but not in others, suggesting (1) that the respective domains may be differently affected by training, and (2) influences other than the (intensive) training alone may have led to these changes. One reason for the difference among the domains can be associated with qualitative differences between the domains and the items within them.

Regarding the potential influences other than manualized training alone, it is important to consider the learning process of the physicians. Depending on how the physician presently communicates, what is important for him/her in the moment of the training and the interaction with the trainer during the training may be different for other physicians in the “same” training. As discussed above in the ancillary analysis of the data, for the three items where the CG was lower than the IG before the training, the CG and the IG reached the same level after the coaching. In all three cases, it seems that the trainers and the trainees concentrated on domains that needed more attention (e.g. structure and checking understanding) before they could move forward to other domains, e.g., dealing with emotions, an important topic for the physicians. In fact, between t0 and t2 (after the coaching), the IG shows a positive (but not significant) change in both items of dealing with emotions (domain C: 2.36 pts (t0); 2.64 pts (t2); see S5 Table), while the CG shows a very small change (2.29 pts (t0); 2.34 pts (t2)). More time and intensive training seems to have been required for the IG to achieve a significant change and for the CG to achieve a change at all. We tried to account for the individuality of learning goals of the physicians by recording the learning goals with a goal attainment scale at the beginning of the workshop. However, the learning goals often changed during the learning process, so that this method, at least as we used it, was too static.

These observations imply that training programs have to be flexible in order to address the different needs of the physicians. Moreover, in the development process of the training concepts it should be taken into account, that different skills/domains may need different intensity of work to be changed, probably dependent on the prior knowledge/skills of the physician. A study by Bylund and colleagues [40] describes, for example, that although no transfer was observed in the trained physicians as a whole, the subgroup of the weaker physicians at baseline showed significant changes on their communicative behavior. Here also arises a further challenge for future research: only a better understanding of the learning processes [44] and the learning context [39,45] of the physicians will allow an enhancement of the training methods.

The two review articles on CSTs [2,7] discussed five RCTs similar to the present study, where actual consultations between health care professionals and patients were evaluated using external raters [34,37,59–61]. Two RCTs [60,61] were concerned with emotion and empathy only, and all RCTs used an interaction analysis method as the main assessment tool. Interaction analysis methods are based on the assumption that certain categories of reactions are good, independently from the context of the consultation [62]. Our study, in contrast, used rating scales, which are less objective but evaluate the performance and appropriateness of the physician’s reactions [57]. Thus, this study not only replicates the heterogeneous results of the other studies but also expands the discussion to further aspects, such as the structure of consultation and the meaning of the context. Moreover, It leads the discussion further to the problem of the limitations of the rating instruments [63–65], a very challenging question for future research.

### Strengths and limitations

Our study has several strengths. The demanding assessment of actual consultations in several areas of oncological medicine assures high external validity. Furthermore, the intervention is based on the literature recommendations [5,55] and incorporates the elements of modern pedagogy [53] and psychology [52]. The additive design used in the present study enabled the evaluation of one component of the training, the coaching, providing information on the specific value of the coaching as an add-on for the workshop.

Our study has some limitations as well. First, the sample calculation was based on a previous study, which showed effect sizes larger than those actually observed in the present study. To verify a hypothesis with such an effect size, twice as many physicians would be necessary. Additionally, it was not possible to complete a video-recorded follow-up a few months after coaching was completed, so our study does not provide information on the long-term effects of this training. Furthermore, as there was no group without intervention, it was not possible to calculate the true effect of the workshop alone.

Second, selection bias may be present, as (1) the participation was voluntary and (2) the patients were partially chosen by the physicians themselves. The effect of involving physicians more interested in communication in our workshop may have been reduced by the possibility of obtaining a certificate that most of the physicians needed for their specialization. Another systematic bias may come from the willingness (or not) of the physicians to have their consultations recorded on video. In fact, the recording prevented many physicians from participating in the present study. It is likely that physicians were influenced not only by their own preferences but also by the work climate and expectations of the departments they worked in [cf. 45]. With respect to patient recruitment, although selection bias cannot be ruled out, the assessment of the patients’ distress suggests that bias was limited, as very distressed patients were asked and agreed to participate in the study. Interestingly, the most distressed patients were those in the IG at t3. It seems that the physicians in this group were more confident in having difficult consultations recorded. The data on the self-evaluation of the physicians is currently being prepared for publication.

Third, our main instrument was not equally reliable in all items, as discussed above [57]. We addressed this issue by using the rater random effect as a covariate for the calculation of the mixed models. Another problem we faced was the daily constraints that did not allow us to strictly follow our study design. As part of their specialization, the physicians regularly rotate from one ward/ambulance to another, which means that some physicians suddenly did not work in oncology. Our solution to this problem was to wait until the next opportunity the physicians had oncological patients again and continue the study from there. Our protocol restricted the training to oncologists as our preliminary work was also restricted to this area. The effects of the training in other medical specialties need to be tested.

### Generalizability

One of greatest strengths of the present study is its high level of external validity and generalizability. The training and assessment were conducted under actual conditions with actual patients.

### Conclusion

In summary, our study suggests that individual coaching is an important add-on for communication skills workshops, and more time and more intensive coaching are needed to achieve significant results, especially in more complex domains, such as dealing with emotions. The small effect sizes reiterated that hard work is required to change behavior. Considering the efforts made in the last years to increase the effect sizes of CSTs, one cannot disregard the fact that learning new behavior requires time and practice that cannot be provided by short training alone. The work with detailed analysis of video recordings of actual consultations seems effective and was well accepted by the physicians.

Indeed, research on communication in medicine is experiencing a turning point. In this context, the present study represents an important contribution: it integrates the key recommendations of experts in communication skills training regarding time, didactics, set up and training [47] for the optimization of didactics and improvement of effect sizes. Thus, these results are promising, despite some statistical limitations, as they show a high degree of external validity and offer new insights for future research.

## Supporting information

S1 Table Evaluation of the consultations at t0 and t1. Evaluation of the consultations (all items and domains) by external raters at t0 and t1 (scale range: 0–4); p-value from paired t-test to assess differences between t0 and t1.
(DOCX)

S2 Table Comparison group difference IG minus CG at t1, baseline t0. Comparison group difference IG minus CG, evaluation of the consultations (all items and domains) by external raters at t1, adjusted in mixed regression models for baseline t0, rater, and patient distress. Parameter Estimates refer to the group difference IG minus CG at t1.
(DOCX)

S3 Table Evaluation of the consultations at t1 and t2. Evaluation of the consultations (all items and domains) by external raters at t1 and t2 (scale range: 0–4); p-value from paired t-test to assess differences between t1 and t2.
(DOCX)

S4 Table Comparison group difference IG minus CG at t2, baseline t1. Comparison group difference IGxCG, evaluation of the consultations (all items and domains) by external raters at t2, adjusted in mixed regression models for baseline t1, rater, and patient distress. Parameter Estimates refer to the group difference IG minus CG at t2.
(DOCX)

S5 Table Evaluation of the consultations at t0 and t2. Evaluation of the consultations (all items and domains) by external raters at t0 and t2 (scale range: 0–4); p-value from paired t-test to assess differences between t0 and t2.
(DOCX)

S6 Table Comparison group difference IG minus CG at t2, baseline t0. Comparison group difference IGxCG, evaluation of the consultations (all items and domains) by external raters at t2, adjusted in mixed regression models for baseline t0, rater, and patient distress. Parameter Estimates refer to the group difference IG minus CG at t2.
(DOCX)

S1 File ComOn-Coaching—Physicians sample. (SAV)

S2 File ComOn-Coaching—Patient sample. (SAV)

S3 File ComOn-Coaching—Consultation ratings. (SAV)

## Abbreviations

CST: communication skills training
ES: effect size
HCP: heath care professional
PPC: physician-patient communication
RCT: randomized controlled trial
