# Correction: ComOn-Coaching: The effect of a varied number of coaching sessions on transfer into clinical practice following communication skills training in oncology: Results of a randomized controlled trial

**DOI:** 10.1371/journal.pone.0211752

**Published:** 2019-01-29

**Authors:** Marcelo Niglio de Figueiredo, Lorena Krippeit, Gabriele Ihorst, Heribert Sattel, Carma L. Bylund, Andreas Joos, Jürgen Bengel, Claas Lahmann, Kurt Fritzsche, Alexander Wuensch

There is an error in the second sentence under the heading “Results” in the Abstract. The correct sentence is: The intervention group showed a statistically significant improvement (ES d = 0.45, p = .01) in the *All items* domain of the rating scales between t1 and t2 and showed a significant advantage compared with the CG (ES d = .41, p = .04).

In Table 7, the values in the column with the heading “Effect Size” are incorrect. Please see the corrected Table 7 here.

**Table 7:** Effect of the coaching on the groups.

Variable (Domain)	Group	Mean t1 (SD)	Mean t2 (SD)	Diff (SD)	Effect Size	P-value
**A1 Start of**	**IG**	**1.98 (0.72)**	**2.27 (0.75)**	**0.29 (0.85)**	**0.34**	**0.0482**
**Conversation**	**CG**	**1.79 (0.60)**	**2.07 (0.70)**	**0.28 (0.79)**	**0.35**	**0.0416**
**A2 Assessing Patient’s**	**IG**	**2.22 (0.86)**	**2.64 (0.75)**	**0.42 (0.82)**	**0.51**	**0.0044**
**Perspective**	CG	2.19 (0.92)	2.14 (0.93)	-.06 (1.06)	-.06	0.7545
**B Structure of**	IG	2.34 (0.60)	2.47 (0.84)	0.14 (0.74)	0.19	0.2782
**Conversation**	**CG**	**2.15 (0.56)**	**2.42 (0.68)**	**0.27 (0.76)**	**0.36**	**0.0368**
**C Emotional Issues**	IG	0.50 (0.67)	2.64 (0.74)	0.14 (0.78)	0.18	0.2938
	CG	2.43 (0.83)	2.34 (0.79)	-.08 (0.83)	-.10	0.5496
**D End of Conversation**	IG	2.02 (0.83)	2.02 (0.87)	0.00 (1.05)	0.00	1.0000
	CG	1.84 (0.66)	1.87 (0.85)	0.03 (1.07)	0.03	0.8768
**E General**	**IG**	**2.67 (0.49)**	**2.84 (0.38)**	**0.18 (0.45)**	**0.40**	**0.0266**
**Communication skills**	CG	2.61 (0.41)	2.61 (0.47)	0.00 (0.43)	0.00	0.9691
**F Overall Evaluation**	IG	2.60 (0.59)	2.69 (0.67)	0.09 (0.72)	0.13	0.4569
	CG	2.44 (0.61)	2.49 (0.79)	0.05 (0.79)	0.06	0.7150
**All items**	**IG**	**2.44 (0.43)**	**2.62 (0.40)**	**0.18 (0.40)**	**0.45**	**0.0102**
	CG	2.35 (0.36)	2.40 (0.49)	0.05 (0.45)	0.11	0.5110

Evaluation of the consultations by external raters at t1 and t2 (scale range: 0–4) separately by treatment group; p-value from paired t-test to assess differences between t1 and t2

## References

[1] Niglio de Figueiredo M, Krippeit L, Ihorst G, Sattel H, Bylund CL, Joos A, et al (2018) ComOn-Coaching: The effect of a varied number of coaching sessions on transfer into clinical practice following communication skills training in oncology: Results of a randomized controlled trial. PLoS ONE 13(10): e0205315 10.1371/journal.pone.0205315 30289905 PMC6173449

