# Supplementary material for: ComOn-Coaching: The effect of a varied number of coaching sessions on transfer into clinical practice following communication skills training in oncology: Results of a randomized controlled trial
Source: PLoS One. 2018 Oct 5;13(10):e0205315. doi: 10.1371/journal.pone.0205315 (PMC6173449; doi:10.1371/journal.pone.0205315)
Supplement: S1 Table — Evaluation of the consultations (all items and domains) by external raters at t0 and t1 (scale range: 0–4); p-value from paired t-test to assess differences between t0 and t1. (DOCX) [file pone.0205315.s001.docx]

| **Variable** | **Group** | **Mean t0 (SD)** | **Mean t1 (SD)** | **Diff (SD)** | **P** |
| --- | --- | --- | --- | --- | --- |
| **A1 Start of** | **IG** | **1.78 (0.69)** | **1.99 (0.72)** | **0.21 (0.86)** | **0.1476** |
| **Consultation** | **CG** | **1.68 (0.66)** | **1.79 (0.60)** | **0.11 (0.70)** | **0.3461** |
|  | ***All*** | ***1.73 (0.67)*** | ***1.89 (0.67)*** | ***0.16 (0.78)*** | ***0.0825*** |
| **A2 Assessing Patient’s** | **IG** | **2.30 (0.90)** | **2.21 (0.85)** | **-0.09 (0.77)** | **0.4605** |
| **Perspective** | **CG** | **2.23 (0.86)** | **2.19 (0.92)** | **-0.03 (1.11)** | **0.8527** |
|  | ***All*** | ***2.27 (0.88)*** | ***2.20 (0.88)*** | ***-0.07 (0.95)*** | ***0.5599*** |
| **B Structure of** | **IG** | **2.36 (0.72)** | **2.32 (0.61)** | **-0.04 (0.86)** | **0.7758** |
| **Consultation** | **CG** | **2.07 (0.53)** | **2.15 (0.56)** | **0.07 (0.76)** | **0.5687** |
|  | ***All*** | ***2.22 (0.65)*** | ***2.24 (0.59)*** | ***0.02 (0.81)*** | ***0.8711*** |
| B1 Active | IG | 2.80 (0.81) | 2.77 (0.62) | -0.02 (1.02) | 0.9050 |
| structuring | CG | 2.51 (0.57) | 2.55 (0.62) | 0.04 (0.67) | 0.7111 |
|  | *All* | *2.65 (0.71)* | *2.66 (0.63)* | *0.01 (0.86)* | *0.9193* |
| B2 Setting | IG | 1.92 (0.88) | 1.88 (0.77) | -0.05 (1.06) | 0.7871 |
| sub-sections | CG | 1.64 (0.67) | 1.74 (0.71) | 0.10 (1.07) | 0.5616 |
|  | *All* | *1.78 (0.79)* | *1.81 (0.73)* | *0.03 (1.06)* | *0.8254* |
| **C Emotional Issues** | **IG** | **2.39 (0.84)** | **2.52 (0.67)** | **0.13 (0.90)** | **0.3934** |
|  | **CG** | **2.29 (0.73)** | **2.43 (0.83)** | **0.14 (0.94)** | **0.3824** |
|  | ***All*** | ***2.34 (0.78)*** | ***2.47 (0.75)*** | ***0.13 (0.92)*** | ***0.2171*** |
| C1 Recognizing | IG | 2.18 (0.82) | 2.28 (0.73) | 0.11 (0.98) | 0.5065 |
| emotions | CG | 2.01 (0.80) | 2.24 (0.86) | 0.24 (0.99) | 0.1609 |
|  | *All* | *2.09 (0.81)* | *2.26 (0.79)* | *0.17 (0.98)* | *0.1397* |
| C2 Offering emotional | IG | 2.61 (0.91) | 2.76 (0.68) | 0.15 (0.93) | 0.3358 |
| support | CG | 2.57 (0.74) | 2.61 (0.90) | 0.04 (1.01) | 0.8052 |
|  | *All* | *2.59 (0.83)* | *2.68 (0.79)* | *0.10 (0.96)* | *0.3970* |
| **D End of Consultation** | **IG** | **1.79 (0.78)** | **1.98 (0.83)** | **0.20 (1.03)** | **0.2530** |
|  | **CG** | **1.69 (0.65)** | **1.84 (0.67)** | **0.14 (0.99)** | **0.3972** |
|  | ***All*** | ***1.74 (0.72)*** | ***1.91 (0.76)*** | ***0.17 (1.00)*** | ***0.1532*** |
| **E General** | **IG** | **2.59 (0.47)** | **2.68 (0.49)** | **0.08 (0.45)** | **0.2755** |
| **communication** | **CG** | **2.47 (0.33)** | **2.61 (0.41)** | **0.13 (0.50)** | **0.1181** |
| ***skills*** | ***All*** | ***2.53 (0.41)*** | ***2.64 (0.45)*** | ***0.11 (0.47)*** | ***0.0563*** |
| E1 clear and | IG | 3.19 (0.53) | 3.31 (0.82) | 0.12 (0.89) | 0.4138 |
| appropriate words | CG | 3.15 (0.71) | 3.38 (0.65) | 0.22 (0.83) | 0.1189 |
|  | *All* | *3.17 (0.62)* | *3.34 (0.74)* | *0.17 (0.86)* | *0.0936* |
| E2 non-verbal | IG | 3.36 (0.58) | 3.47 (0.57) | 0.10 (0.53) | 0.2484 |
| communication | CG | 3.18 (0.58) | 3.34 (0.60) | 0.16 (0.79) | 0.2465 |
|  | *All* | *3.27 (0.58)* | *3.40 (0.58)* | *0.13 (0.67)* | *0.1041* |
| E3 pacing and | IG | 2.87 (0.85) | 2.95 (0.81) | 0.07 (0.85) | 0.5961 |
| making pauses | CG | 2.71 (0.82) | 2.97 (0.65) | 0.26 (0.98) | 0.1152 |
|  | *All* | *2.79 (0.84)* | *2.96 (0.73)* | *0.17 (0.91)* | *0.1207* |
| E4 offering to | IG | 2.86 (0.97) | 2.93 (0.82) | 0.07 (0.96) | 0.6414 |
| ask questions | CG | 2.90 (0.74) | 2.72 (0.70) | -0.19 (0.94) | 0.2375 |
|  | *All* | *2.89 (0.86)* | *2.83 (0.77)* | *-0.05 (0.95)* | *0.6244* |
| E5 checking | IG | 0.66 (0.85) | 0.76 (0.99) | 0.10 (1.04) | 0.5589 |
| understanding | CG | 0.42 (0.53) | 0.61 (0.66) | 0.19 (0.78) | 0.1593 |
|  | *All* | *0.54 (0.72)* | *0.68 (0.84)* | *0.14 (0.92)* | *0.1856* |
| **F Overall Evaluation** | **IG** | **2.55 (0.86)** | **2.60 (0.58)** | **0.05 (0.85)** | **0.7019** |
|  | **CG** | **2.40 (0.60)** | **2.44 (0.61)** | **0.04 (0.80)** | **0.7567** |
|  | ***All*** | ***2.48 (0.73)*** | ***2.52 (0.60)*** | ***0.05 (0.82)*** | ***0.6195*** |
| **All items** | **IG** | **2.40 (0.44)** | **2.45 (0.43)** | **0.05 (0.40)** | **0.4908** |
|  | **CG** | **2.24 (0.34)** | **2.35 (0.36)** | **0.11 (0.48)** | **0.1729** |
|  | ***All*** | ***2.32 (0.40)*** | ***2.40 (0.39)*** | ***0.08 (0.44)*** | ***0.1334*** |
