# Supplementary material for: ComOn-Coaching: The effect of a varied number of coaching sessions on transfer into clinical practice following communication skills training in oncology: Results of a randomized controlled trial
Source: PLoS One. 2018 Oct 5;13(10):e0205315. doi: 10.1371/journal.pone.0205315 (PMC6173449; doi:10.1371/journal.pone.0205315)
Supplement: S3 Table — Evaluation of the consultations (all items and domains) by external raters at t1 and t2 (scale range: 0–4); p-value from paired t-test to assess differences between t1 and t2. (DOCX) [file pone.0205315.s003.docx]

| **Variable** | **Group** | **Mean t1 (SD)** | **Mean t2 (SD)** | **Diff (SD)** | **P** |
| --- | --- | --- | --- | --- | --- |
| **A1 Start of** | **IG** | **1.98 (0.72)** | **2.27 (0.75)** | **0.29 (0.85)** | **0.0482** |
| **Consultation** | **CG** | **1.79 (0.60)** | **2.07 (0.70)** | **0.28 (0.79)** | **0.0416** |
|  | ***All*** | **1.88 (0.66)** | **2.17 (0.73)** | **0.29 (0.81)** | ***0.0042*** |
| **A2 Assessing Patient’s** | **IG** | **2.22 (0.86)** | **2.64 (0.75)** | **0.42 (0.82)** | **0.0044** |
| **Perspective** | **CG** | **2.19 (0.92)** | **2.14 (0.93)** | **-0.06 (1.06)** | **0.7545** |
|  | ***All*** | ***2.21 (0.89)*** | ***2.39 (0.88)*** | ***0.18 (0.97)*** | ***0.1186*** |
| **B Structure of** | **IG** | **2.34 (0.60)** | **2.47 (0.84)** | **0.14 (0.74)** | **0.2782** |
| **Consultation** | **CG** | **2.15 (0.56)** | **2.42 (0.68)** | **0.27 (0.76)** | **0.0368** |
|  | ***All*** | ***2.24 (0.59)*** | ***2.45 (0.71)*** | ***0.20 (0.75)*** | ***0.0226*** |
| B1 Active | IG | 2.78 (0.63) | 2.85 (0.83) | 0.08 (0.81) | 0.5731 |
| structuring | CG | 2.55 (0.62) | 2.89 (0.71) | 0.34 (0.87) | 0.0251 |
|  | *All* | *2.66 (0.63)* | *2.87 (0.77)* | *0.21 (0.84)* | *0.0399* |
| B2 Setting | IG | 1.90 (0.77) | 2.09 (0.81) | 0.19 (0.95) | 0.2280 |
| sub-sections | CG | 1.74 (0.71) | 1.95 (0.84) | 0.21 (0.93) | 0.1879 |
|  | *All* | *1.82 (0.74)* | *2.02 (0.82)* | *0.20 (0.93)* | *0.0715* |
| **C Emotional Issues** | **IG** | **0.50 (0.67)** | **2.64 (0.74)** | **0.14 (0.78)** | **0.2938** |
|  | **CG** | **2.43 (0.83)** | **2.34 (0.79)** | **-0.08 (0.83)** | **0.5496** |
|  | ***All*** | ***2.47 (0.75)*** | ***2.49 (0.77)*** | ***0.03 (0.81)*** | ***0.7711*** |
| C1 Recognizing | IG | 2.27 (0.74) | 2.47 (0.77) | 0.19 (0.76) | 0.1342 |
| emotions | CG | 2.24 (0.86) | 2.12 (0.89) | -0.13 (0.95) | 0.4341 |
|  | *All* | *2.26 (0.79)* | *2.29 (0.85)* | *0.03 (0.87)* | *0.7354* |
| C2 Offering emotional | IG | 2.74 (0.68) | 2.82 (0.84) | 0.08 (0.93) | 0.5949 |
| support | CG | 2.61 (0.90) | 2.57 (0.81) | -0.04 (0.89) | 0.7813 |
|  | *All* | *2.67 (0.79)* | *2.69 (0.83)* | *0.02 (0.91)* | *0.8463* |
| **D End of Consultation** | **IG** | **2.02 (0.83)** | **2.02 (0.87)** | **0.00 (1.05)** | **1.0000** |
|  | **CG** | **1.84 (0.66)** | **1.87 (0.85)** | **0.03 (1.07)** | **0.8768** |
|  | ***All*** | ***1.93 (0.75)*** | ***1.94 (0.85)*** | ***0.01 (1.05)*** | ***0.9097*** |
| **E General** | **IG** | **2.67 (0.49)** | **2.84 (0.38)** | **0.18 (0.45)** | **0.0266** |
| **communication** | **CG** | **2.61 (0.41)** | **2.61 (0.47)** | **0.00 (0.43)** | **0.9691** |
| ***skills*** | ***All*** | ***2.64 (0.45)*** | ***2.73 (0.44)*** | ***0.09 (0.45)*** | ***0.0951*** |
| E1 clear and | IG | 3.46 (0.61) | 3.31 (0.83) | 0.15 (0.97) | 0.3722 |
| appropriate words | CG | 3.37 (0.65) | 3.36 (0.55) | -0.01 (0.72) | 0.9088 |
|  | *All* | *3.34 (0.74)* | *3.41 (0.58)* | *0.07 (0.85)* | *0.5131* |
| E2 non-verbal | IG | 3.43 (0.59) | 3.43 (0.52) | 0.00 (0.65) | 1.0000 |
| communication | CG | 3.33 (0.59) | 3.16 (0.82) | -0.17 (0.75) | 0.1931 |
|  | *All* | *3.38 (0.59)* | *3.30 (0.70)* | *-0.08 (0.70)* | *0.3190* |
| E3 pacing and | IG | 2.93 (0.81) | 3.09 (0.73) | 0.16 (1.03) | 0.3586 |
| making pauses | CG | 2.97 (0.65) | 2.84 (0.77) | -0.13 (0.78) | 0.3445 |
|  | *All* | *2.95 (0.73)* | *2. 97 (0.76)* | *0.02 (0.92)* | *0.8732* |
| E4 offering to | IG | 2.90 (0.81) | 3.23 (0.64) | 0.33 (0.76) | 0.0144 |
| ask questions | CG | 2.72 (0.70) | 2.77 (0.92) | 0.06 (0.80) | 0.6794 |
|  | *All* | 2.81 (0.76) | 3.00 (0.82) | 0.19 (0.79) | *0.0431* |
| E5 checking | IG | 0.77 (1.00) | 1.02 (0.87) | 0.26 (0.79) | 0.0589 |
| understanding | CG | 0.61 (0.66) | 0.90 (0.82) | 0.28 (1.01) | 0.1005 |
|  | *All* | *0.69 (0.85)* | *0.96 (0.85)* | *0.27 (0.90)* | *0.0130* |
| **F Overall Evaluation** | **IG** | **2.60 (0.59)** | **2.69 (0.67)** | **0.09 (0.72)** | **0.4569** |
|  | **CG** | **2.44 (0.61)** | **2.49 (0.79)** | **0.05 (0.79)** | **0.7150** |
|  | ***All*** | ***2.52 (0.60)*** | ***2.59 (0.74)*** | ***0.07 (0.75)*** | ***0.4359*** |
| **All items** | **IG** | **2.44 (0.43)** | **2.62 (0.40)** | **0.18 ( 0.40)** | **0.0102** |
|  | **CG** | **2.35 (0.36)** | **2.40 (0.49)** | **0.05 (0.45)** | **0.5110** |
|  | ***All*** | ***2.40 (0.43)*** | ***2.51 (0.46)*** | ***0.12 (0.43)*** | ***0.0257*** |
