# Supplementary material for: ComOn-Coaching: The effect of a varied number of coaching sessions on transfer into clinical practice following communication skills training in oncology: Results of a randomized controlled trial
Source: PLoS One. 2018 Oct 5;13(10):e0205315. doi: 10.1371/journal.pone.0205315 (PMC6173449; doi:10.1371/journal.pone.0205315)
Supplement: S4 Table — Comparison group difference IGxCG, evaluation of the consultations (all items and domains) by external raters at t2, adjusted in mixed regression models for baseline t1, rater, and patient distress. Parameter Estimates refer to the group difference IG minus CG at t2. (DOCX) [file pone.0205315.s004.docx]

|  | **Group difference IG minus CG** | | | | |
| --- | --- | --- | --- | --- | --- |
| **Variable** | **Estimate** | **95% CI** | **Effect Size** | **Stand. Error** | **P** |
| **A1 Start of Consultation** | **0.16** | **-0.15 to 0.47** | **0.19** | **0.16** | **0.3029** |
| **A2 Assessing Patient’s Perspective** | **0.50** | **0.13 to 0.86** | **0.51** | **0.19** | **0.0084** |
| **B Structure of Consultation** | **-0.04** | **-0.36 to 0.29** | **-0.04** | **0.16** | **0.8223** |
| B1 Active structuring | -0.07 | -0.44 to 0.30 | -0.07 | 0.19 | 0.7177 |
| B2 Setting sub-sections | 0.04 | -0.31 to 0.39 | 0.04 | 0.18 | 0.8261 |
| **C Emotional Issues** | **0.26** | **-0.07 to 0.59** | **0.28** | **0.17** | **0.1273** |
| C1 recognizing emotions | 0.28 | -0.09 to 0.66 | 0.29 | 0.19 | 0.1359 |
| C2 offering emot. Support | 0.23 | -0.13 to 0.59 | 0.23 | 0.18 | 0.2011 |
| **D End of Consultation** | **0.10** | **-0.31 to 0.51** | **0.10** | **0.21** | **0.6305** |
| **E General Communication Skills** | **0.25** | **0.07 to 0.44** | **0.54** | **0.10** | **0.0063** |
| E1 clear and appropriate words | 0.18 | -0.06 to 0.42 | 0.24 | 0.12 | 0.1401 |
| E2 non-verbal communication | 0.24 | -0.04 to 0.53 | 0.32 | 0.14 | 0.0935 |
| E3 pacing and making pauses | 0.35 | 0.03 to 0.67 | 0.34 | 0.16 | 0.0338 |
| E4 offering to ask questions | 0.40 | 0.07 to 0.73 | 0.49 | 0.17 | 0.0189 |
| E5 checking understanding | 0.04 | -0.03 to 0.39 | 0.04 | 0.18 | 0.8249 |
| **F Overall Evaluation** | **0.13** | **-0.20 to 0.47** | **0.15** | **0.17** | **0.4247** |
| **All items** | **0.19** | **0.00 to 0.38** | **0.41** | **0.10** | **0.0446** |

**S4 Table**: Comparison group difference IGxCG, evaluation of the consultations (all items and domains) by external raters at t2, adjusted in mixed regression models for baseline t1, rater, and patient distress. Parameter Estimates refer to the group difference IG minus CG at t2
