# Supplementary material for: ComOn-Coaching: The effect of a varied number of coaching sessions on transfer into clinical practice following communication skills training in oncology: Results of a randomized controlled trial
Source: PLoS One. 2018 Oct 5;13(10):e0205315. doi: 10.1371/journal.pone.0205315 (PMC6173449; doi:10.1371/journal.pone.0205315)
Supplement: S5 Table — Evaluation of the consultations (all items and domains) by external raters at t0 and t2 (scale range: 0–4); p-value from paired t-test to assess differences between t0 and t2. (DOCX) [file pone.0205315.s005.docx]

| **Variable** | **Group** | **Mean t0 (SD)** | **Mean t2 (SD)** | **Diff (SD)** | **P** |
| --- | --- | --- | --- | --- | --- |
| **A1 Start of** | **IG** | **1.79 (0.70)** | **2.27 (0.75)** | **0.48 (0.72)** | **0.0004** |
| **Consultation** | **CG** | **1.68 (0.66)** | **2.07 (0.70)** | **0.39 (0.89)** | **0.0126** |
|  | ***All*** | ***1.74 (0.68)*** | ***2.17 (0.73)*** | ***0.43 (0.80)*** | ***<0.0001*** |
| **A2 Assessing Patient’s** | **IG** | **2.33 (0.90)** | **2.64 (0.75)** | **0.31 (1.02)** | **0.0750** |
| **Perspective** | **CG** | **2.23 (0.86)** | **2.14 (0.93)** | **-0.09 (1.19)** | **0.6521** |
|  | ***All*** | ***2.28 (0.88)*** | ***2.39 (0.88)*** | ***0.11 (1.12)*** | ***0.4029*** |
| **B Structure of** | **IG** | **2.39 (0.72)** | **2.47 (0.74)** | **0.08 (1.06)** | **0.6391** |
| **Consultation** | **CG** | **2.07 (0.53)** | **2.42 (0.68)** | **0.35 (0.82)** | **0.0153** |
|  | ***All*** | ***2.23 (0.65)*** | ***2.45 (0.71)*** | ***0.22 (0.95)*** | ***0.0578*** |
| B1 Active | IG | 2.80 (0.82) | 2.85 (0.83) | 0.06 (1.27) | 0.7946 |
| structuring | CG | 2.51 (0.57) | 2.89 (0.71) | 0.38 (0.90) | 0.0154 |
|  | *All* | *2.65 (0.71)* | *2.87 (0.77)* | *0.22 (1.11)* | *0.0975* |
| B2 Setting | IG | 1.97 (0.86) | 2.09 (0.81) | 0.13 (1.07) | 0.4893 |
| sub-sections | CG | 1.64 (0.67) | 1.95 (0.84) | 0.31 (0.94) | 0.0530 |
|  | *All* | *1.80 (0.78)* | *2.02 (0.82)* | *0.22 (1.00)* | *0.0687* |
| **C Emotional Issues** | **IG** | **2.36 (0.83)** | **2.64 (0.74)** | **0.28 (1.09)** | **0.1307** |
|  | **CG** | **2.29 (0.73)** | **2.34 (0.79)** | **0.06 (0.82)** | **0.6859** |
|  | ***All*** | ***2.32 (0.77)*** | ***2.49 (0.78)*** | ***0.17 (0.96)*** | ***0.1425*** |
| C1 Recognizing | IG | 2.15 (0.82) | 2.47 (0.77) | 0.31 (1.11) | 0.0993 |
| emotions | CG | 2.01 (0.80) | 2.12 (0.89) | 0.11 (0.89) | 0.4565 |
|  | *All* | *2.08 (0.80)* | *2.29 (0.85)* | *0.21 (1.00)* | *0.0767* |
| C2 Offering emotional | IG | 2.57 (0.90) | 2.82 (0.84) | 0.25 (1.19) | 0.2155 |
| support | CG | 2.57 (0.74) | 2.57 (0.81) | 0.00 (0.93) | 1.0000 |
|  | *All* | *2.57 (0.82)* | *2.69 (0.83)* | *0.13 (1.07)* | *0.3233* |
| **D End of Consultation** | **IG** | **1.75 (0.79)** | **2.02 (0.87)** | **0.27 (1.04)** | **0.1372** |
|  | **CG** | **1.69 (0.65)** | **1.82 (0.81)** | **0.13 (1.06)** | **0.4777** |
|  | ***All*** | ***1.72 (0.72)*** | ***1.92 (0.84)*** | ***0.20 (1.05)*** | ***0.1180*** |
| **E General** | **IG** | **2.59 (0.47)** | **2.84 (0.38)** | **0.26 (0.48)** | **0.0025** |
| **communication** | **CG** | **2.47 (0.33)** | **2.61 (0.47)** | **0.14 (0.55)** | **0.1488** |
| ***skills*** | ***All*** | ***2.53 (0.41)*** | ***2.73 (0.44)*** | ***0.20 (0.52)*** | ***0.0018*** |
| E1 clear and | IG | 3.18 (0.53) | 3.46 (0.61) | 0.28 (0.69) | 0.0204 |
| appropriate words | CG | 3.15 (0.71) | 3.36 (0.55) | 0.21 (0.73) | 0.0971 |
|  | *All* | *3.17 (0.62)* | *3.41 (0.58)* | *0.24 (0.71)* | *0.0047* |
| E2 non-verbal | IG | 3.34 (0.58) | 3.44 (0.53) | 0.10 (0.49) | 0.2387 |
| communication | CG | 3.18 (0.58) | 3.16 (0.83) | -0.01 (0.80) | 0.9165 |
|  | *All* | *3.26 (0.58)* | *3.30 (0.71)* | *0.04 (0.66)* | *0.5899* |
| E3 pacing and | IG | 2.84 (0.84) | 3.09 (0.73) | 0.25 (1.11) | 0.1846 |
| making pauses | CG | 2.71 (0.82) | 2.85 (0.77) | 0.14 (1.01) | 0.4140 |
|  | *All* | *2.77 (0.83)* | *2.97 (0.76)* | *0.19 (1.05)* | *0.1217* |
| E4 offering to | IG | 2.85 (0.98) | 3.23 (0.64) | 0.38 (1.03) | 0.0350 |
| ask questions | CG | 2.90 (0.74) | 2.77 (0.92) | -0.13 (1.05) | 0.4558 |
|  | *All* | *2.88 (0.86)* | *3.00 (0.82)* | *0.12 (1.06)* | *0.3346* |
| E5 checking | IG | 0.67 (0.86) | 1.03 (0.87) | 0.35 (0.98) | 0.0368 |
| understanding | CG | 0.42 (0.53) | 0.89 (0.82) | 0.47 (1.03) | 0.0096 |
|  | *All* | *0.55 (0.72)* | *0.96 (0.85)* | *0.41 (1.00)* | *0.0008* |
| **F Overall Evaluation** | **IG** | **2.53 (0.86)** | **2.68 (0.67)** | **0.15 (0.96)** | **0.3482** |
|  | **CG** | **2.40 (0.60)** | **2.49 (0.79)** | **0.09 (0.87)** | **0.5387** |
|  | ***All*** | ***2.47 (0.74)*** | ***2.59 (0.74)*** | ***0.12 (0.91)*** | ***0.2628*** |
| **All items** | **IG** | **2.40 (0.44)** | **2.62 (0.40)** | **0.23 (0.47)** | **0.0064** |
|  | **CG** | **2.24 (0.34)** | **2.40 (0.49)** | **0.16 (0.58)** | **0.1050** |
|  | ***All*** | ***2.32 (0.40)*** | ***2.51 (0.46)*** | ***0.19 (0.53)*** | **0.0026** |
